# ICA II Alleviates Testicular Torsion Injury by Dampening the Oxidative and Inflammatory Stress

**DOI:** 10.3389/fendo.2022.871548

**Published:** 2022-05-12

**Authors:** Ani Chi, Bicheng Yang, Xiaohui Cao, Zhenqing Wang, Hanchao Liu, Hao Dai, Chunhua Deng, Min Zhang

**Affiliations:** ^1^Department of Andrology, The First Affiliated Hospital, Sun Yat-sen University, Guangzhou, China; ^2^Department of Parasitology of Zhongshan School of Medicine, Sun Yat-sen University, Guangzhou, China; ^3^Hubei Key Laboratory for Kidney Disease Pathogenesis and Intervention, Hubei Polytechnic University, School of Medicine, Huangshi, China; ^4^School of Materials Science and Engineering, South China University of Technology, Guangzhou, China

**Keywords:** testicular torsion, icariside II, testosterone, fertility, oxidative stress, inflammatory stress

## Abstract

Testicular torsion-detorsion is an ischaemia-reperfusion-induced male gonad injury that may lead to male infertility. Oxidative stress plays an important role in the ischaemia-reperfusion injury. Icariside II (ICA II) prevents oxidative stress and has obvious protective effects on spermatogenic function. The present study was aimed to investigate therapeutic potentials of ICA II on testicular torsion. 72 mice were randomly divided into three groups: sham-operated control group (n = 24), testicular ischemia-reperfusion + saline group (n = 24) and testicular ischemia-reperfusion + icariside II treated group (n = 24). Testicular ischemia-reperfusion was induced by the left testis rotated 360 degrees in a clockwise direction for 30 minutes followed by detorsion, the contralateral testis was removed. ICA II in saline (5 mg/kg/day) was administrated by gavage immediately after detorsion. The results demonstrated that ICA II alleviated testicular damage by mitigating spermatogenic cell injury and improving testosterone production in mouse models of testicular torsion. We revealed that ICA II alleviated oxidative stress and apoptosis in the testes, reduced inflammatory infiltration and accelerated angiogenesis. Briefly, ICA II administration ameliorated testicular damage by improving spermatogenic function and testosterone production, which supports its use as a pharmacological treatment of testicular torsion.

## Introduction

Testicular torsion is the most common cause of acute paediatric scrotal disease and it can occur at any age (1). The torsion duration and degree of twisting of the spermatic cord are the most important factors determining the severity of testicular damage (2). Testicular torsion causes apoptosis of germ cells (3) and decreases sperm production (4). The hormonal function of the testes is also affected. Torsion results in reduced testosterone production of Leydig cells (5), which supports spermatogenesis (6).

Testicular-torsion induced damage is a typical ischemia-reperfusion injury. During reperfusion, oxygen is supplied to the testis, which leads to the production of reactive oxygen species (ROS) (7), causing endothelial damage and germinal cell apoptosis (8). Endogenous molecules are released into the extracellular space, particularly in the context of cell damage and activate an immune response (9). Resident macrophages play important roles in the initiation of inflammatory responses, and depletion of resident macrophages reduces monocyte and neutrophil influx. An inflammatory response after ischaemia and reperfusion causes the subsequent generation of more ROS (10) and an increase in the expression of proinflammatory cytokines. ROS and inflammation activate apoptotic pathways and lead to germ cell-specific apoptosis (11). Many pharmacological agents with antioxidant, anti-inflammatory or ROS scavenging properties were successfully studied in animal models (12). However, no clinical benefits of these treatments were reported.

Icariside II (ICA II. C 27 H 32° 10, MW 514.54) is the bioactive form of icariside I, which is isolated from Herba Epimedii (13–15). ICA II has been traditionally used in clinical practice for over 2000 years in East Asian countries. Previous studies showed that ICA II had beneficial effects on diabetic erectile dysfunction (ED) (16–18). Its therapeutic efficacy was linked to the following factors: morphological recovery of tissue injury associated with endothelial dysfunction, smooth muscle atrophy, neuropathy and collagen deposition (19, 20). Notably, ICA II improved the fertility potential of diabetic rats by maintaining normal spermatogenesis *via* antioxidant effects (21). Therefore, we hypothesized that ICA II would be feasible to treat testicular torsion.

The present study evaluated the therapeutic effects of ICA II in a testicular torsion mouse model. We showed that ICA II promoted testis regeneration after testicular torsion *via* the reshaping of local microenvironments by reducing ROS, pro-inflammatory factor levels and inhibiting monocyte and neutrophils. We demonstrated that ICA II effectively relieved ischaemia-reperfusion injury and improved spermatogenesis function. The results of the present study inspire novel perspectives for the application of ICA II in testicular torsion.

## Materials and Methods

### Testicular Torsion Mouse Model

All C57BL/6J mice (male, 8–10 weeks old) were purchased from the Animal Center at the Medical Laboratory of Guangdong Province, and all experimental animal processes were approved by the Animals Care and Used Committee of the First Affiliated Hospital of Sun Yat-sen University (Guangzhou, China). Animals had free access to a standard laboratory diet and water before and after surgery and were maintained in a constant environment of 50% humidity and 20°C with a 12-h dark-light cycle.72 mice were randomly divided into three groups: sham-operated control group (n = 24), testicular ischemia-reperfusion + saline group (n = 24) and testicular ischemia-reperfusion + icariside II treated group (n = 24). Mice were anesthetized by 10% chloral hydrate (3μL/1g). No analgesic was administered. Mice were anesthetized by 10% chloral hydrate (3μL/1g). No analgesic was administered. A standard testicular torsion model was established as previously described (22, 23). The abdomen was opened, and the left testis was rotated 360° in a clockwise direction followed by fixation with silk suture maintained for 30 minutes. The rotated testis was reversed and the contralateral testis was removed. ICA II in saline (5 mg/kg/day) was administrated by gavage immediately after detorsion (n=6 mice per group). The control animals (n=6 mice per group) received saline with the same volume. Normal animals were used as the untreated group. On day 1, 3, 7 and 14, 6 mice per group were sacrificed at each time point and testicular tissue samples were harvested for further analysis.

### Histological Analysis

Testicular tissues were harvested from sacrificed mice on day 14. Tissues were fixed in 4% paraformaldehyde, embedded in paraffin and cut into 5-µm sections. The sections were dewaxed, rehydrated, and repaired with ethylenediamine tetra acetic acid. Testicular tissue sections were blocked for 60 min with 5% BSA then incubated with primary antibodies overnight at 4°C. Sections were washed with PBS three times and incubated with secondary antibodies for 60 min at room temperature in the dark. DAPI was used for 5 min to stain nuclei. The following antibodies were used: anti-F4/80 (Abcam, ab100790, 1:400), anti-Ki67 (Abcam, ab15580, 1:400), anti-SOX9 (Abcam, ab185966, 1:100), and anti-VASA (Abcam, ab270534, 1:100), anti-SCP3 (Abcam, ab97672, 1:100), anti-CD31 (Abcam, ab182981, 1:100), anti-α-SMA (Abcam, ab5694, 1:100), Alexa Fluor 488-conjugated goat anti-rabbit (Abcam, ab150077, 1:500), Alexa Fluor 488-conjugated goat anti-mouse (Abcam, ab150113, 1:500), Alexa Fluor 594-conjugated goat anti-rabbit (Abcam, ab150080, 1:500). Alexa Fluor 594-conjugated goat anti-mouse (Abcam, ab150116, 1:500), DAPI (Abcam, ab228549, 1:1000). Images were acquired using a confocal microscope (LSM800, Zeiss). To investigate the testis structure, Sections of 5 μm thickness were stained with haematoxylin and eosin-phloxine solution.

### Assessment of ROS Levels in the Testes

ROS are indicators of the level of oxidative stress and may be used to assess the oxidative environment of the testes. ROS production was assessed using a 2′,7′-dichlorodihydrofluorescein diacetate (Beyotime, S0063, 10 μmol/L) histochemical assay. Nuclei were stained with DAPI, and images were captured under a microscope (LSM800, Zeiss). The fluorescence intensity of the staining was measured using ImageJ.

### RNA Isolation and RT–qPCR

Total RNA was purified from testicular tissue samples using Nucleozol reagent (Macherey-Nagel, 740404.200) according to the manufacturer’s protocol. Total RNA was reverse transcribed into cDNA using the Prime Script RT kit (TaKaRa, E237). Quantitative PCR (qPCR) was performed using SYBR mix (ChamQ, Q311-02) according to the manufacturer’s instructions. Signals were detected using a Bio–Rad CFX96 detection system (Roche). The primers that were used for this study are outlined in **Table 1**.

**Table 1:** Primers used to amplify transcripts during RT-qPCR analysis.

Gene		Primers	TM	Length(bp)
*Gapdh*	Forward Primer	AGGTCGGTGTGAACGGATTTG	62.6	21
	Reverse Primer	GGGGTCGTTGATGGCAACA	62.6	19
*Il-1β*	Forward Primer	ATGATGGCTTATTACAGTGGCA A	61.3	23
	Reverse Primer	GTCGGAGACGTAGCTGGA	61.8	18
*Cyp11a1*	Forward Primer	AGGTCCTTCAATGAGATCCCTT	60.2	22
	Reverse Primer	TCCCTGTAAATGGGGCCATAC	61.3	21
*Cyp17a1*	Forward Primer	GCCCAAGTCAAAGACACCTAAT	60.2	23
	Reverse Primer	GTACCCAGGCGAAGAGAATAGA	60.7	23

Relative gene expression was calculated using the 2^-ΔΔCT^ method.

Cytokine levels measurement.

Testicular tissues were lysed in ice-cold RIPA lysis buffer (Elabscience®, E-BC-R327) and the supernatants were collected. The supernatants protein concentrations were normalized using the BCA assay. Protein levels were analyzed using a LEGEND plex bead-based immunoassay (BioLegend, 740134) according to the manufacturer’s instructions. The mean fluorescence intensity of the cytokines was analyzed using LEGEND plex8.0 software.

Testicular tissues were lysed in ice-cold RIPA lysis buffer (Elabscience^®^, E-BC-R327) and the supernatants were collected. The supernatants protein concentrations were normalized using the BCA assay. Protein levels were analyzed using a LEGEND plex bead-based immunoassay (BioLegend, 740134) according to the manufacturer’s instructions. The mean fluorescence intensity of the cytokines was analyzed using LEGEND plex8.0 software.

### Flow Cytometric Analysis

Testicular tissues were cut into small pieces using micro scissors followed by the addition of collagenase type IV (Stem cell Technologies, 07909, 1 mg/mL) and digested in water at 37°C for 15 min. The digestion was terminated *via* the addition of PBS containing 10% BSA. The homogenate was filtered through a 50-μm filter and centrifuged at 1200 g for 3 min at 4°C. Cell precipitates were resuspended in PBS to obtain a mouse testicular cell suspension. For flow cytometry analysis of macrophages, monocytes and neutrophils, cells were incubated with antibodies diluted 1:100 at 4°C for 20 min, and antibodies were used as follows: CD45-PE-Cyanine7 (eBioscience™, 4277984, 1:100), F4/80-Brilliant Violet 421 (BioLegend, 123131, 1:100), CD11b-FITC (Abcam, ab24874, 1:100), Ly6G-APC (Thermo Fisher, 17-9668-82, 1:100) and Ly6C-APC (Abcam, ab93550, 1:100). For flow cytometry analysis of Leydig cell, cells were incubated with primary antibodies diluted 1:100 at 4°C for 20 min, cells were washed with PBS three times and incubated with secondary antibodies diluted 1:100 at 4°C for 20 min in the dark, and antibodies were used as follows: Anti-LH Receptor (Alomone Labs, ALR-010, 1:100) and Alexa Fluor 488-conjugated goat anti-rabbit (BioLegend, 406416, 1:100). For detection of cell apoptosis, PI and Annexin V-APC staining liquid (Elabscience^®^, E-CK-A217) was added, and the cells were sorted using flow cytometry after avoiding light for 10 min to obtain the results of flow analysis. Fluorescence-activated cell sorting (FACS) was performed using MoFlo Astrios EQs (Beckman Coulter).

### Western Blotting

Testicular tissues or cells were lysed in ice-cold RIPA lysis buffer (Elabscience^®^, E-BC-R327) containing phosphatase inhibitor cocktail (Abcam, ab201111) with 1 mM PMSF (Thermo Fisher, 36978) for 30 min. Lysates were centrifuged at 12,000 g for 15 min, and the supernatant was collected for protein determination. The concentration of total protein was measured using a BCA protein assay kit (Abcam, ab102536). Protein was prepared in SDS sample buffer, subjected to SDS–PAGE then transferred to a nitrocellulose membrane. The PVDF membranes were blocked in 5% non-fat powdered milk and incubated with primary antibodies diluted 1:1000 in blocking buffer. Bands were detected using the enhanced chemiluminescence (ECL) method (MerckMillipore, WBKLS0050). The following primary antibodies were used: anti-β-tubulin (AAB, A41015-2, 1:1000), anti-caspase3 (Cell Signalling Technology, 9662S, 1:1000), anti-cleaved-caspase3 (Cell Signalling Technology, 9664T, 1:1000).

### Measurement of Hormone Levels

Blood samples were collected from the tail vein of mice, and the samples were centrifuged (1000 g, 15 min) to obtain the serum. Testosterone levels were measured using an enzyme-linked immunosorbent assay (ELISA) kit (Abcam, ab108666). The absorbance of testosterone was measured at 405 nm.

### Statistical Analysis

Statistical analyses were performed using one-way ANOVA in GraphPad Prism version 8. Quantification of fluorescence intensity was performed using ImageJ. Data are expressed as the means ± SD for at least 3 experiments. *P*<0.05 was considered statistically significant.

## Results

### ICA II Protected Spermatogenic Function in Testicular Torsion of Mouse Model

**Figure 1:**
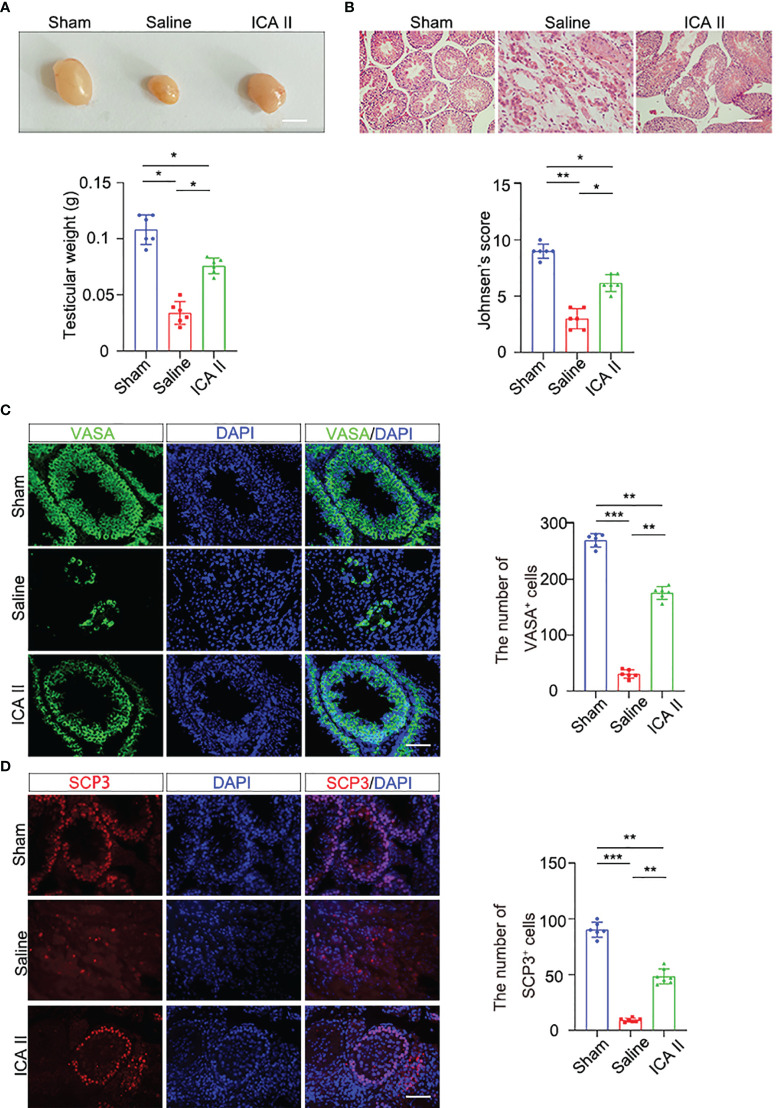
ICA II alleviated spermatogenic cell injury during testicular torsion and detorsion. **(A)** Representative images of testes from the normal and testicular torsion group (treated with saline, ICA II) 14 days after testicular torsion and quantification of testes weight; n=6 per group, Scale bars=5 mm. **(B)** Histopathological analyses of testes obtained from the normal and testicular torsion group (treated with saline, ICA II), and Johnsen’s score were evaluated 14 days after testicular torsion. n=6 per group, Scale bars=100 µm. **(C)** Immunostaining of mouse seminiferous tubules using the germ cell marker VASA 14 days after torsion and quantification of seminiferous tubules containing VASA^+^ cells. n=6 per group, Scale bars=100 µm. **(D)** Immunostaining of testicular tissues with SCP3 14 days after torsion and quantification of seminiferous tubules containing SCP3^+^ cells. n=6 per group, Scale bars=100 µm. Data are presented as the mean ± SD. *p < 0.05, **p < 0.01, ***p < 0.001.

To investigate whether ICA II attenuated testicular torsion injury, testes were collected 14 days after ICA II treatment. ICA II treatment significantly restored testis weights (**Figure 1A**). HE staining showed that the sham-operated animals had intact germinal epithelium. The structure of the seminiferous tubules was disrupted and the number of germ cells decreased significantly in the saline group. ICA II preserved spermatogenic function in testicular torsion-detorsion mouse model of ischaemia/reperfusion injury. Johnsen’s score was significantly higher in ICA II-treated group compared to the saline group (**Figure 1B**). Sertoli cells is essential for spermatogenesis (24). To verify whether ICA II had a beneficial effect on the maintenance of the spermatogenesis environment, we examined the expression of the Sertoli cell marker SOX9 (25, 26). SOX9^+^ cells virtually disappeared in the saline group compared to the sham group, and the number of SOX9^+^ cells increased significantly in the seminiferous tubules after ICA II treatment compared to the saline group (**Figure S1A**). VASA is a marker of germ cells, and VASA staining revealed a decrease in the proportions of VASA^+^ cells at day14 in the saline group. The number of VASA^+^ cells was less severely reduced in the seminiferous tubules in the ICA II-treated group. VASA^+^ cells were more abundant in the seminiferous tubules of the ICA II-treated group than the saline group (**Figure 1C**). To determine whether ICA II protected recipient spermatogenesis, we analysed the expression of Ki67, which is a marker that reflects the proliferation of spermatogonia. Images showed that seminiferous tubules of Ki67^+^ cells virtually decreased in the saline group compared to the sham group, and the number of Ki67^+^ cells increased significantly in the ICA II-treated group compared to the saline group (**Figure S1B**). We examined the expression of the meiotic marker synaptonemal complex protein 3 (SYCP3). SYCP3^+^ cells nearly disappeared in the saline group compared to the sham-operated group (**Figure 1D**), and the number of SYCP3^+^ cells increased significantly in the ICA II-treated group compared to the saline group. These results suggest that ICA II contributed to the protection of spermatogenesis.

### ICA II Maintained Testosterone Production

**Figure 2:**
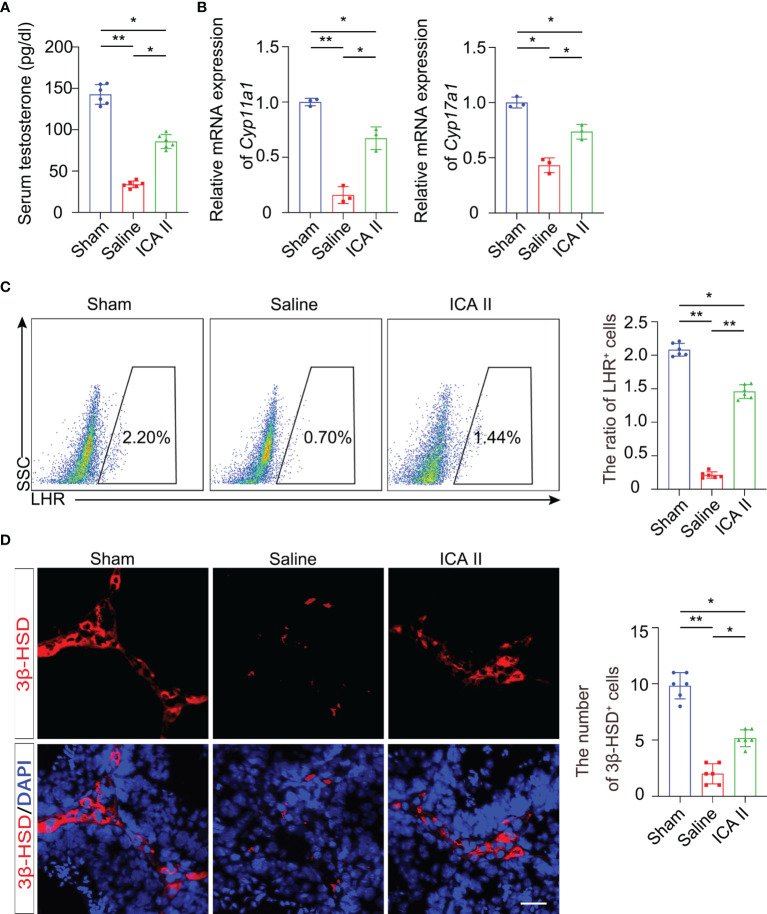
ICA II maintained production of testosterone. **(A)** ELISA analysis of testosterone in serum; n=6 per group. **(B)** The mRNA levels of *Cyp11a1* and *Cyp17a1* in whole testes 14 days after testicular torsion were analysed; n=3 per group. **(C)** Representative flow cytometry of LHR^+^ cells in the testes 14 days after detorsion and quantitative analysis of the frequency of LHR^+^ cells; n=6 per group. **(D)** Immunostaining of testicular tissues with 3β-HSD 14 days after torsion and quantification of 3β-HSD^+^ cells. n=6 per group, Scale bars=100 µm. Data are presented as the mean ± SD. *p < 0.05, **p < 0.01, ***p < 0.001.

Reproduction is dependent on the balance of testosterone (27, 28). Testosterone is primarily produced in Leydig cells. To investigate whether ICA II had beneficial effects on the maintenance of testosterone, we examined testosterone secretion in mice 14 days after testicular torsion. Testicular torsion reduced serum testosterone levels on day 14, and ICA II significantly rescued the testicular torsion-induced low testosterone (**Figure 2A**). ICA II significantly increased the mRNA expression levels of Leydig cell specific markers, *Cyp11a1* and *Cyp17a1* (**Figure 2B**). Flow cytometry revealed that testicular torsion reduced the number of Leydig cells on day14 and ICA II significantly rescued the ratio of Leydig cells (**Figure 2C**). Leydig cells gate selection was presented (**Figure S2A**). We observed that 3β-HSD^+^ cells were rarely observed in the interstitium in the saline group. However, ICA II treatment dramatically increased the number of Leydig cells (**Figure 2D**). These results suggest that ICA II promotes Leydig cell regeneration and testosterone synthesis.

### ICA II Reduced Testicular Torsion Injury-Induced Oxidative Stress and Apoptosis

**Figure 3:**
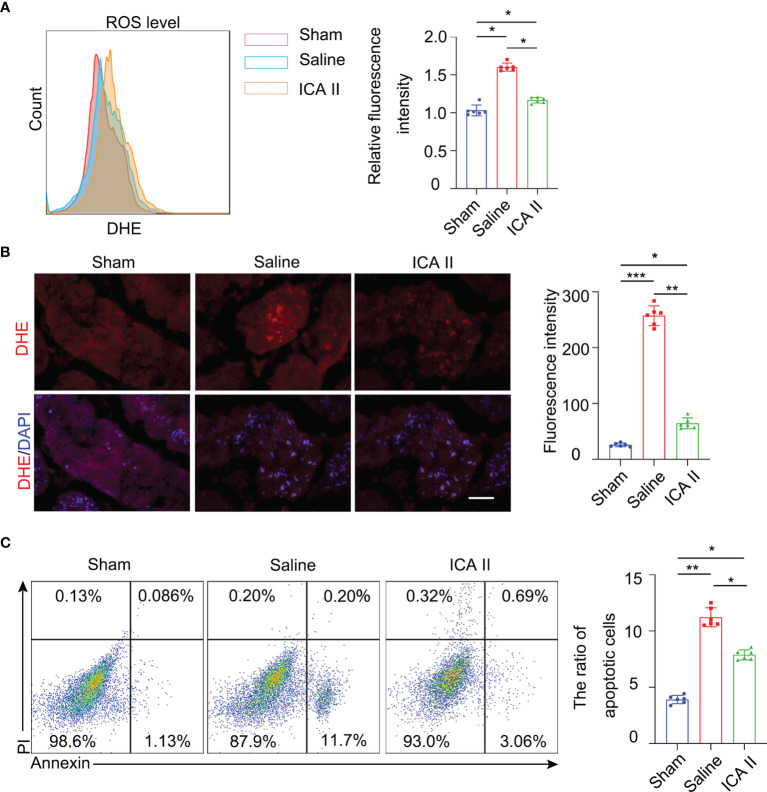
ICA II reduced ROS production and protected against cell apoptosis. **(A)** Representative flow cytometry profiles showing the level of ROS in the testes at 1 day after detorsion and quantification of the relative mean fluorescence intensity; n=6 per group. **(B)** Immunostaining of testicular tissues on day 1 using the ROS probe DHE and quantification of the fluorescence intensity of ROS in every seminiferous tubule; n=6 per group. Scale bar=100 µm. **(C)** Representative flow cytometry profiles showing the impact of treatment with ICA II on the survival of germ cells at 1 day after detorsion. Flow cytometry-based quantification of apoptotic cells in the testes of each group; n=6 per group. Data are presented as the mean ± SD. *p < 0.05, **p < 0.01, ***p < 0.001.

Previous studies showed that ICA II improved the activity of antioxidant enzymes in STZ-induced diabetic rats, which produced ROS (29). The present study examined whether ICA II reduced acute oxidative damage. We analysed the level of ROS in testes using flow cytometry on day 1. ROS were excessively produced during ischaemia-reperfusion injury in saline group. However, ROS levels from the ICA II-treated group were significantly lower than the saline group 1 day after testicular torsion (**Figure 3A**). IF staining showed that ICA II treatment downregulated ROS production (**Figure 3B**). ROS triggers apoptosis *via* the intrinsic pathway. Germ cell apoptosis was significantly reduced in the ICA II group compared to the saline group (**Figure 3C**). Western blot analysis showed that the levels of cleaved caspase 3 in the ICA II group were lower than the saline group (**Figure S3A**). Taken together, these results indicate that the anti-ROS and anti-apoptotic effects of ICA II on the testes play an important role in protecting against testicular torsion injury.

### ICA II Suppressed the Inflammatory Response by Reducing Proinflammatory Immune Monocytes/Macrophages in the Injured Testes

**Figure 4:**
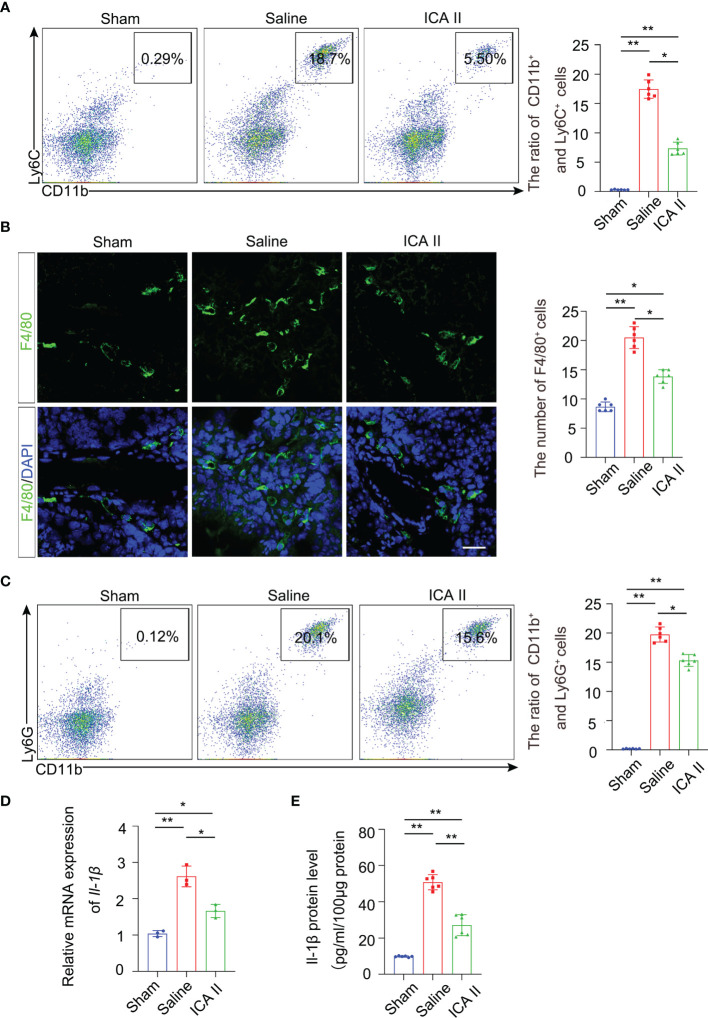
ICA II limited testicular torsion injury-induced inflammation. **(A)** The percentages of CD11b^+^ Ly6C^+^ monocytes in the testes were analysed using flow cytometry 3 days after testicular torsion. Flow cytometry-based quantification of the indicated cells in the testes of each group; n=6 per group. **(B)** Immunostaining revealed that fewer F4/80^+^ cells accumulated in the ICA II-treated group than in the saline group. Nuclei were counterstained with DAPI (blue); n=6 per group. Scale bars=100 µm. **(C)** Flow cytometric analyses of CD11b^+^ Ly6G^+^ neutrophils in the testes 3 days after testicular torsion. Flow cytometry-based quantification of the indicated cells in the testes of each group; n=6 per group. **(D)** a Real-time PCR analysis of mRNA levels of Il-1β in testicular tissues at days 3 after torsion; n=3 per group. **(E)** Quantification of the protein levels of Il-1β in testicular tissues at 3 days after torsion; n=6 per group. Data are presented as the mean ± SD. *p < 0.05, **p < 0.01, ***p < 0.001.

Testicular torsion leads to germ cell apoptosis exacerbating the inflammatory response. Therefore, we investigated whether ICA II participated in testicular repair by rapidly and effectively suppressing excessive immune responses during the early inflammation stage. Flow cytometry revealed that the accumulation of CD11b^+^ Ly6C^+^ monocytes (**Figure 4A**) and CD45^+^F4/80^+^ macrophages (**Figure S4A**) were attenuated in the testes of ICA II-treated mice 3 days after testicular torsion compared to the saline group. IF staining analysis showed that the number of F4/80^+^ macrophages was greatly reduced in the ICA II-treated group 3 days after testicular torsion (**Figure 4B**). These data suggest that treatment with ICA II significantly reduced the total number of pro-inflammatory immune monocytes/macrophages in the injured testicular tissue after testicular torsion. Flow cytometry revealed that ICA II-treated mice showed diminished accumulation of CD11b^+^ Ly6G^+^ neutrophils in the testes at day 3 compared to the saline group (**Figure 4C**). Monocytes, macrophages and neutrophils gates selection was presented (**Figure S4B**). To further assess the effects of ICA II treatment on the testicular torsion environment, we performed qPCR analysis of genes involved in inflammation. The analyses were performed using whole testes tissue collected on day 3 of the ICA II treatment and saline group. The pro-inflammatory cytokine IL-1β was increased in saline group mice, the gene expression (**Figure 4D**) and protein level (**Figure 4E**) of IL-1β were suppressed in the ICA II-treated mice. These data indicate that ICA II significantly suppresses the inflammatory response.

### ICA II Ameliorated Microvascular Dysfunction

**Figure 5:**
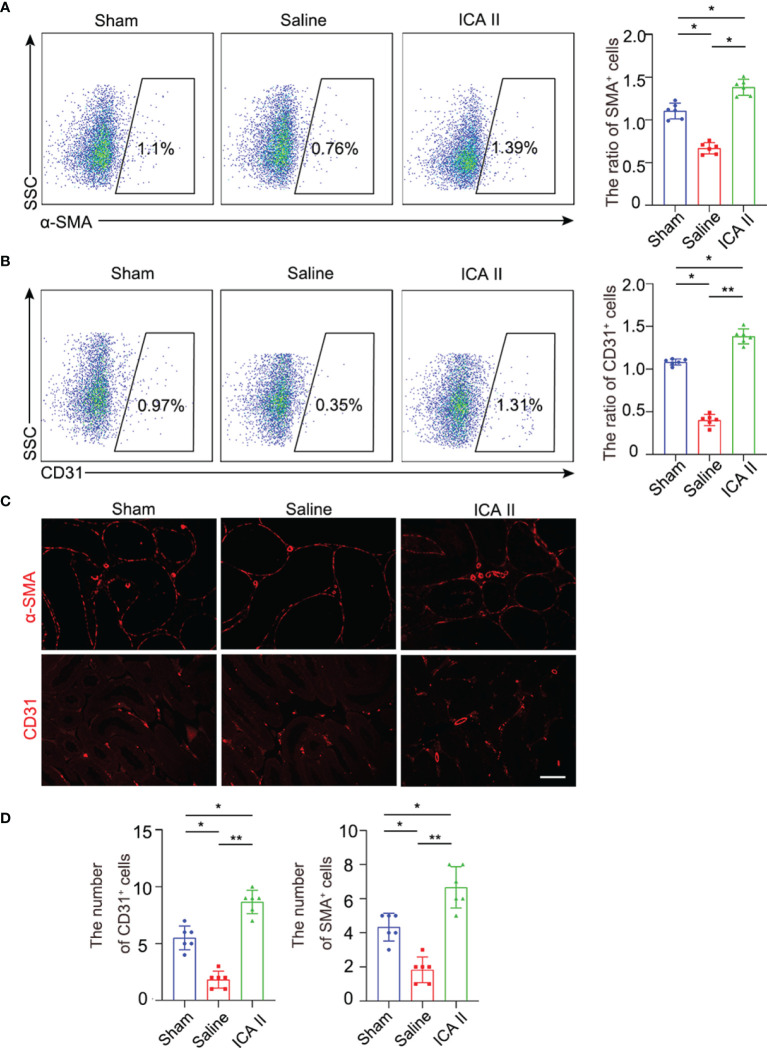
ICA II promoted vascular recovery. **(A)** The percentages of α-SMA+ cells in the testes were analysed using flow cytometry 7 days after testicular torsion. Flow cytometry-based quantification of the indicated cells in the testes of each group; n=6 per group. **(B)** The percentages of CD31^+^ in the testes were analysed using flow cytometry 7 days after testicular torsion. Flow cytometry-based quantification of the indicated cells in the testes of each group; n=6 per group. **(C)** Representative images of different experimental group showing α-SMA^+^ vascular smooth muscle and CD31^+^ endothelial cells. **(D)** Quantification of the number of α-SMA^+^ vascular smooth muscle and CD31^+^ cells. Data are presented as the mean±SD. *p < 0.05, **p < 0.01.

ICA II has a protective role in promoting endothelial cell proliferation and alleviating smooth muscle atrophy in diabetic ED (30). We examined whether ICA II alleviated endothelial injury in testicular torsion. Flow cytometry revealed that α-SMA^+^ cells were lower in the testes of the saline group compared to sham group at day7, and the number of α-SMA^+^ cells increased significantly in the ICA II-treated group compared to the sham group (**Figure 5A**) smooth muscle gate selection was presented (**Figure S5A**). Flow cytometry found that CD31^+^ cells were lower in the saline group compared to the sham group in the testes at day7, and the number of CD31^+^ cells increased significantly in the ICA II-treated group compared to the sham group (**Figure 5B**). Endothelial cell gate selection was presented (**Figure S5B**). Immunofluorescence staining was used to evaluate the effect of ICA II on testis microvasculature. Similarly, the numbers of α-SMA^+^ and CD31^+^ cells were higher in the ICA II-treated group (**Figures 5C, D**). These results suggest that the therapeutic effects of ICA II promote the processes of vasculogenesis.

## Discussion

Testicular torsion involves rotation of the testis and twisting of the spermatic cord, which may lead to testicular atrophy. Early ischaemia-reperfusion injury leads to oxidative stress the generation of large amounts of ROS and cellular apoptosis in tissues. A previous study showed that ICA II decreased tissue heat stress and oxidative stress in C. elegans (31). The present study established a testicular torsion mouse model to investigate whether ICA II treatment protected against testicular torsion injury. We found that ICA II reduced ROS, the infiltration of inflammatory cells and promote the regeneration of spermatogenic cells.

Leydig cells in the interstitial space adjacent to the seminiferous tubule produce testosterone. Testosterone is essential for spermatogenesis. We found that testicular torsion reduced serum testosterone level at day 14, and ICA II significantly rescued the testosterone level. Sertoli cells are well known as somatic cells of the spermatic cord epithelium and provide appropriate hormonal and physical support for the development and maturation of spermatogonia (25). Previous studies have shown that ICA II significantly increased the expression of vimentin filaments in STZ-induced diabetic rats, and the collapse of vimentin filaments led to the separation of spermatogonia from the spermatic epithelium and apoptosis, Sertoli cells in rat testes survive and secrete an almost normal proteome after testicular torsion (32, 33). However, the number of Sertoli cells was significantly lower in the saline group than the sham group in the present study. IF staining showed that ICA II treatment increased the number of Sertoli cells compared to the saline group, which confirmed that ICA II had a protective effect on spermatogenic function.

ROS are produced *via* normal metabolic reactions that play a role in various processes, such as pathogen killing and cell signalling (34, 35). The overproduction of ROS is toxic and induces early tissue injuries. Testicular torsion is a pathological event that is primarily characterized by a reduction in blood supply followed by the reestablishment of perfusion, which causes the formation of ROS. Previous studies have shown that ICA II improved the fertility potential of diabetic men by preserving normal sperm production *via* an antioxidant effect. ROS were overproduced during testicular torsion injury on 1 day after testicular torsion in the present study. However, ICA II dramatically reduced the level of ROS, which indicates that ICA II reduces oxidative stress. Apoptosis plays an important role in development and homeostasis, and it is essential for normal spermatogenesis in human (36). However, uncontrolled apoptosis causes pathological tissue damage. Our results demonstrated that ICA II dramatically reduced apoptosis after testicular torsion. Apoptotic pathways are activated by inflammation and ROS during testicular ischaemia-reperfusion injury.

Testicular torsion is a typical paediatric lesion, and it is representative of the ischaemia-reperfusion injuries that are observed in other organs (37, 38). Macrophages are residents of major organs and one of the first responders to local damage. The number of macrophages increase in the kidney within 24 hours of reperfusion in a mouse model of acute kidney injury. We found that the frequency of CD45^+^F4/80^+^ macrophages was significantly lower in the ICA II-treated group than the saline group, as well as monocytes and neutrophils. The production of ROS from necrotic and injured cells contribute to an environment that promotes resident macrophages into pro-inflammatory macrophages. Pro-inflammatory macrophages are responsible for the production of pro-inflammatory cytokines, their increased levels are indirect evidence of testicular injury. ICA II reduced the levels of IL-1β, which suggests that ICA II had a significant effect on the inhibition of inflammation.

Endothelial cells (ECs) are located in the inner wall of blood vessels and act as metabolic gatekeepers by adapting the delivery of oxygen and nutrients to the metabolic needs of tissues (39). The persistent impairment of spermatogenesis after testicular torsion is associated with altered microvascular blood flow in the testes. Here, we found that the number of CD31^+^ and α-SMA^+^ cells was significantly increased in the ICA II-treated group compared to the saline group. ICA II ameliorates endothelial dysfunction by regulating the MAPK pathway in human cavernous endothelial cells exposed to a diabetic-like environment, and ICA II amelioration of erectile function is involved in promoting endogenous stem cell differentiation into smooth muscle cells. We only focused on the influence of ICA II on the testicular environment after testicular torsion. There are many limitations to our research, and the specific molecular mechanisms of ICA II should be further elucidated.

## Conclusions

The present study showed that ICA II protected germ cells from testicular torsion/detorsion-induced injury. ICA II ameliorated acute oxidative damage by reducing ROS levels and protected germ cells from apoptosis after testicular torsion injury. ICA II reduced the inflammatory response, as evidenced by the reduced expression of inflammatory factors and infiltration of pro-inflammatory monocytes/macrophages. ICA II may be a new treatment for testicular torsion injury to promote spermatogenesis.

## Data Availability Statement

The original contributions presented in the study are included in the article/**Supplementary Material**. Further inquiries can be directed to the corresponding authors.

## Ethics Statement

The animal study was reviewed and approved by the Animals Care and Used Committee of the First Affiliated Hospital of Sun Yat-sen University.

## Author Contributions

MZ: Conception and design, manuscript writing, and obtaining financial support. CD: Conception and design and obtaining financial support. AC: Collection and assembly of data, data analysis and interpretation, and manuscript writing. BY: Collection and assembly of data, data analysis and interpretation, manuscript writing and obtaining financial support. XC: Assembly of data and data analysis, manuscript writing and financial support. HL: Collection and assembly of data, data analysis, and interpretation. ZW: Collection and assembly of data. HD: Collection and assembly of data. All authors contributed to the article and approved the submitted version.

## Funding

This work was supported by the National Natural Science Foundation of China (81873829, MZ; 81671449, CD) and the China Postdoctoral Science Foundation (2020M683127, BY) and Scientific Innovation Project of Hubei Polytechnic University (21xjz07A, XC).

## Conflict of Interest

The authors declare that the research was conducted in the absence of any commercial or financial relationships that could be construed as a potential conflict of interest.

## Publisher’s Note

All claims expressed in this article are solely those of the authors and do not necessarily represent those of their affiliated organizations, or those of the publisher, the editors and the reviewers. Any product that may be evaluated in this article, or claim that may be made by its manufacturer, is not guaranteed or endorsed by the publisher.
