# Supplementary material for: ICA II Alleviates Testicular Torsion Injury by Dampening the Oxidative and Inflammatory Stress
Source: Front Endocrinol (Lausanne). 2022 May 12;13:871548. doi: 10.3389/fendo.2022.871548 (PMC9135456; doi:10.3389/fendo.2022.871548)
Supplement: Supplementary file 1 [file DataSheet_1.pdf]

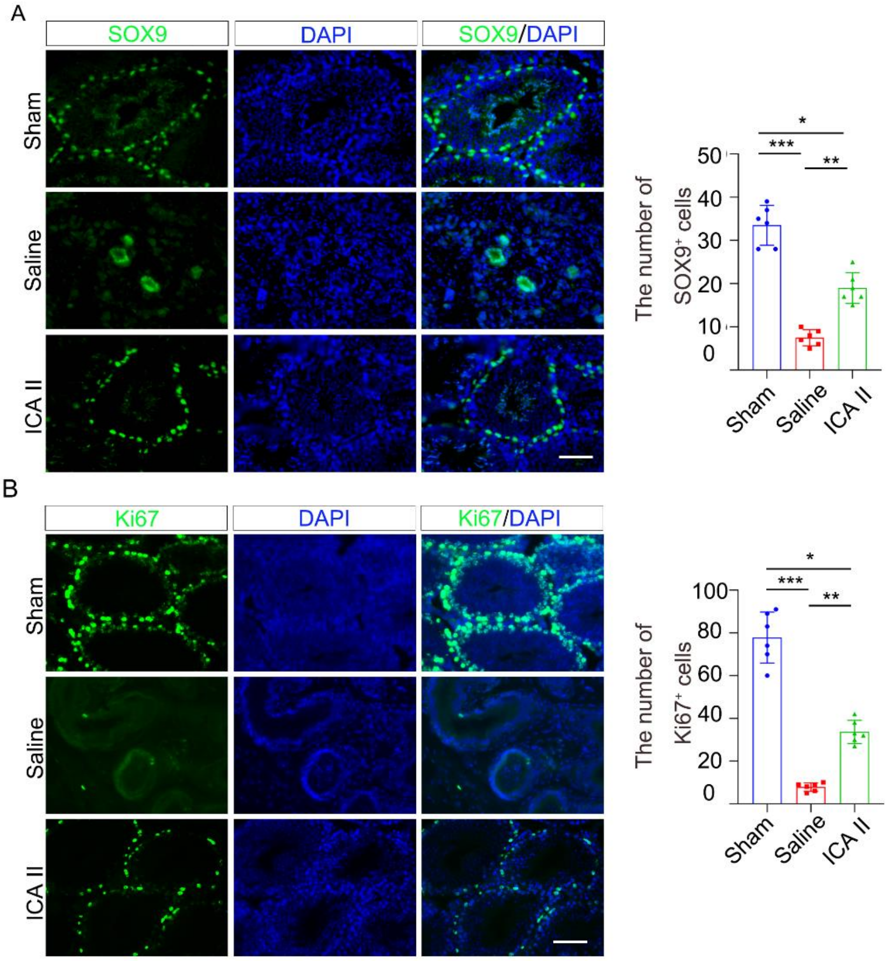
A
SOX9
DAPI
SOX9/DAPI
Sham
Saline
ICA II
The number of SOX9⁺ cells
50
40
30
20
10
0
*
***
**
Sham
Saline
ICA II
B
Ki67
DAPI
Ki67/DAPI
Sham
Saline
ICA II
The number of Ki67⁺ cells
100
80
60
40
20
0
*
***
**
Sham
Saline
ICA II

Supplemental figures

Figure S1: **ICA II restored recipient spermatogenesis.**

A. Immunofluorescence staining of testicular tissues with SOX9 14 days after torsion and quantification of seminiferous tubules containing $SOX9^{+}$ cells. n=6 per group, Scale bars=100 μm. B. Immunostaining of testicular tissues with the proliferation marker Ki67 14 days after torsion and quantification of seminiferous tubules containing $Ki67^{+}$cells. n=6 per group, Scale bars=100 μm. Data are presented as the mean ± SD. * $p < 0.05$, ** $p < 0.01$, *** $p < 0.001$.

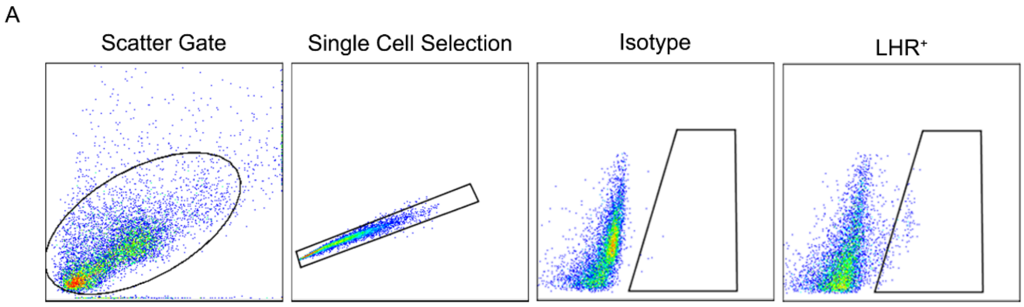

Figure S2: Gate selection for Leydig cells. A. Gate selection for LHR^+ cells.

A

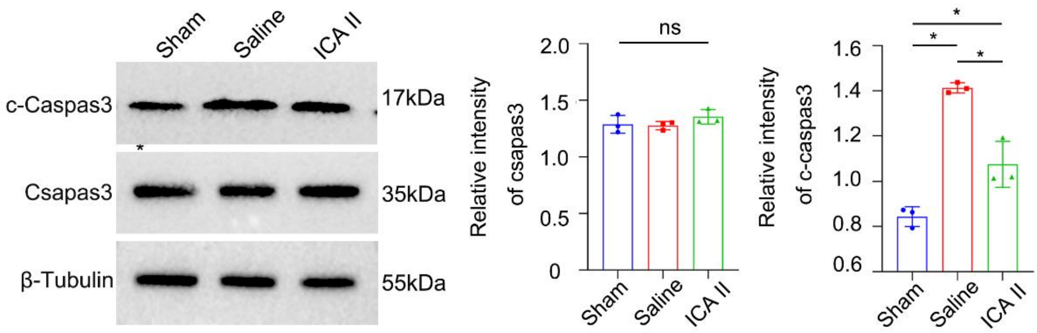

Sham
Saline
ICA II
c-Caspas3
17kDa
Csapas3
35kDa
β-Tubulin
55kDa
Relative intensity of csapas3
ns
2.0
1.5
1.0
0.5
0
Relative intensity of c-caspas3
1.6
1.4
1.2
1.0
0.8
0.6
*

Figure S3: **ICA II reduced germ cell apoptosis**

A Western blot analyses of testicular tissues 1 day after torsion showing significantly decreased cleaved-caspase 3 levels in the ICA II-treated group compared to the saline group. Data are presented as the mean ± SD. * $p < 0.05$, ** $p < 0.01$, *** $p < 0.001$.

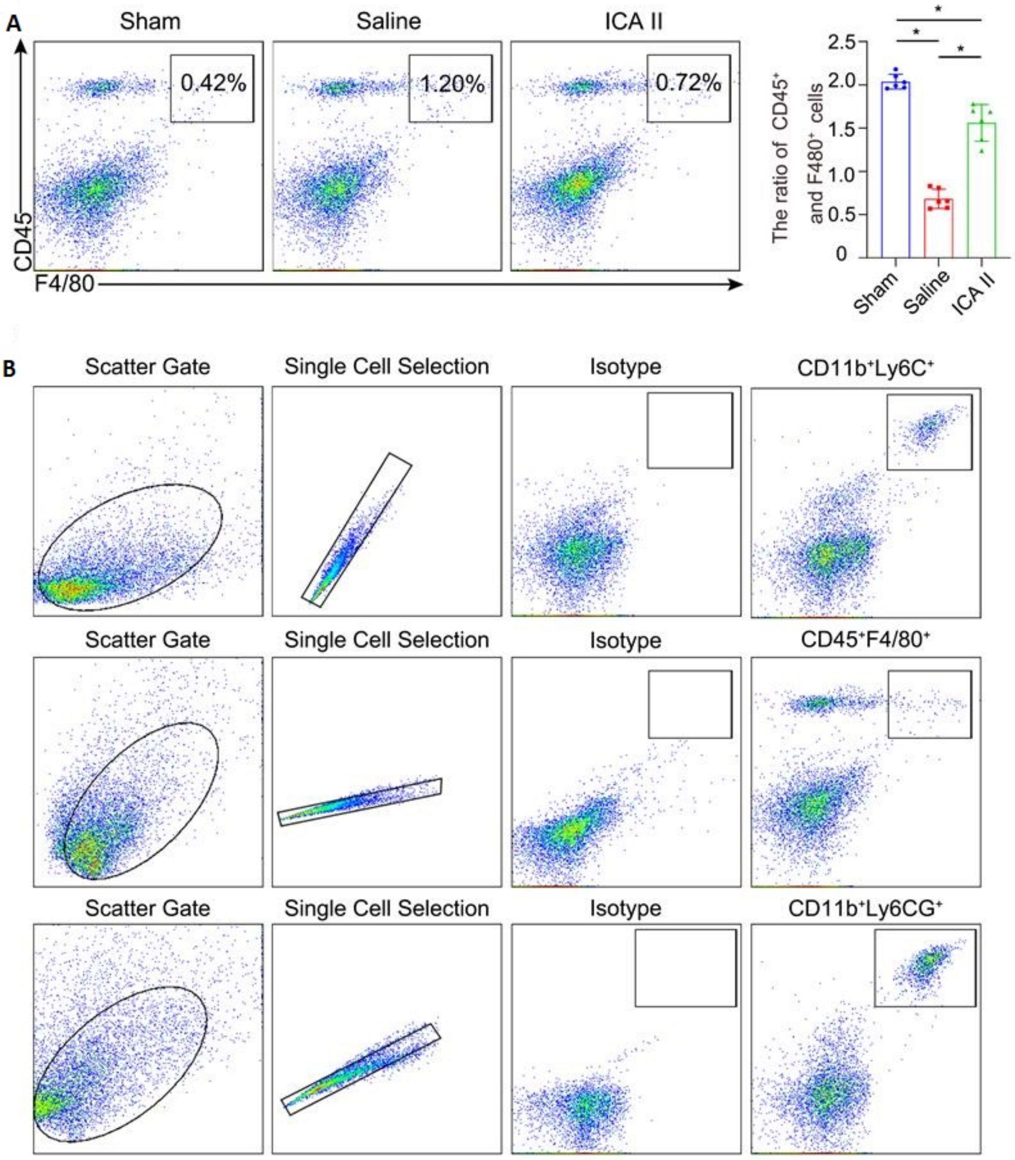
A
Sham
Saline
ICA II
0.42%
1.20%
0.72%
CD45
F4/80
The ratio of CD45+ and F480+ cells
2.5
2.0
1.5
1.0
0.5
0
*
Sham
Saline
ICA II
B
Scatter Gate
Single Cell Selection
Isotype
CD11b+Ly6C+
CD45+F4/80+
CD11b+Ly6CG+

Figure S4: **The inflammatory microenvironment was improved in injured testes.**

A. The percentages of $CD45^{+}F4/80^{+}$ macrophages in the testes were analysed using flow cytometry 3 days after testicular torsion. Flow cytometry-based quantification of the indicated cells in the testes of each group; n=6 per group. B. Monocytes, macrophages and neutrophils gates selection. Data are presented as the mean ±SD. * $p < 0.05$, ** $p < 0.01$, *** $p < 0.001$.

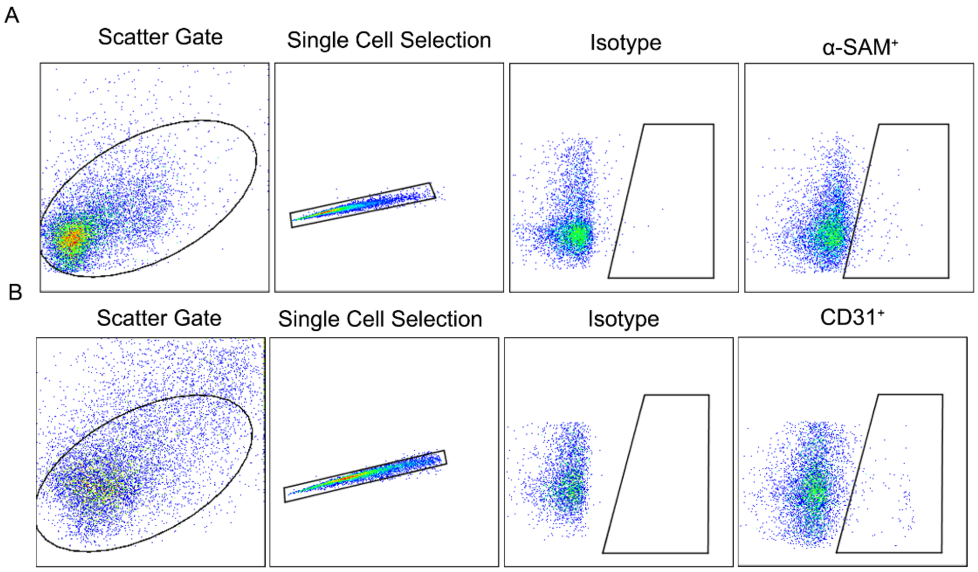
A
Scatter Gate
Single Cell Selection
Isotype
α-SAM⁺
B
Scatter Gate
Single Cell Selection
Isotype
CD31⁺

Figure S5: Gate selection for smooth muscle or endothelial cell

A**.** Gate selection for α-SMA$^+$ cells. B**.** Gate selection for CD31$^+$ cells.
